# Infantile Convulsions

**Published:** 1882-09

**Authors:** O. P. Baer

**Affiliations:** Richmond, Indiana; Bureau Clinical Medicine, I. H. A.


### INFANTILE CONVULSIONS.

O. P. Baer, M. D., Richmond, Ind.

(*Bureau Clinical Medicine, I. H. A.*)

Fellows of the International Hahnemannian Association:—I have selected for my present theme, the subject of *Infantile Convulsions*, for several prominent reasons. The first of which is, because parents, as a general thing, have as great, if not greater innate dread of convulsions among their children, than of any other disease. The second is, because when convulsions do occur, they often seriously disturb both parents and physician. The third is, because spasms are often the antecedents of other diseases; and fourth, because the mortality of children by convulsions, particularly in cities, is very great. The brighter the child, the more active the brain; the more nervous the temperament, the larger the head; the more exciting the surroundings, the greater the danger of convulsions. The more receding the forehead, the smaller the head, the duller the intellect, the more lymphatic the child, the less danger there is of spasms. These are axioms to be recognized by all studious physicians. Convulsions are protean in form, as they often start in highly clonic and terminate atonic. Or in other cases commence atonic and become tonic as they advance. Sometimes they start in with an intense vigor, convulsing every muscle and nerve for several minutes, then relaxing as though the sanitary end was just at hand, when lo, the whole scene is re-enacted over again and again for an untold number of times. Again, they may start in almost unobserved twitching of this muscle, then of that, here and there, marked by fretfulness and feverishness of the infant, soon to be followed by more decided symptoms. These premonitory warnings may not at all be heeded or even noticed by the parents, until the sad scene in all its horrors is upon them; then what confusion, what excitement, what bustle, what running for doctors, and above all, what an utter want of good hard sense is often exhibited by the attendants! Then again, severe cases frequently present themselves in apparently perfect health. The child may be playing in all its accustomed glee and mirthfulness, and in the midst of its fun convulsed from head to foot. Moaning, tossing, jerking, frothing, grating its teeth, rolling its eyes, distorting its mouth and gasping for breath, flushed face, high fever, and worse than all, an excited brain.

Convulsions rarely exert their equal force upon both sides at the same time. The right arm and left leg, or left arm and right leg, or arm and leg of the same side may be convulsed immoderately, while the other parts may lay more or less quiet, or really be paralyzed for the time being, or even permanently so. Spasms frequently pass off but partially, leaving a tremulous weakness of the nerves for days, and an undefinable, inexpressible something in the general physique which is not natural, but not sufficiently characteristic to yield to vocal descriptive language. Other times our little patients may have two, three or more hard convulsions and get right up, as though nothing had happened. Some children have spasms on all unusual occasions, while others never have them at all. The teething period is generally the most trying one of an infant's life. Its whole system is rendered sensitive by the constant growth and upward pressure of the coming teeth upon the gums. While among country and well-nursed babes this natural process seldom gives any annoyance, in cities, and particularly among improperly-fed children, it is much to be dreaded. Worms as a concomitant, add greatly to the strife. The children of some families are peculiarly liable to convulsions, whether from some family idiosyncrasy, or diathesis, or heredity, I am not prepared to say, but the fact of tendency to spasms exists, irrefutably. I treated one large family for more than thirty years, embracing the entire child-bearing period, twelve children in all, and not one of them escaped spasms. There was constant parental anxiety from birth until the mouth was full of teeth. I have a family now who have had four children, one of whom died for me in spasms; the others have all had spasms, frequently lasting thirty minutes at a time. The youngest, now approaching its period of freedom, has had at least one hundred hard spasms in the past two and a half years. The last one held her nearly two hours without intermission, causing all of us to doubt her recovery. I feared disorganization of the brain, but to my great relief she rallied perfectly in every respect, as well as ever, bright, cheerful and happy. Indeed, she is the most precocious of any of the children. The father, when in his seventeenth year, had a prolonged and severe attack of chorea. He developed slowly, showing more or less signs of scrofula. The mother is of a restless type.

Having generalized, I will now individualize a few notable cases, with treatment.

The child referred to above, having had so many spasms, had them at intervals of two, three or four weeks, for the space of nearly two years, sometimes very light, not requiring treatment; then again she would have four or five during twenty-four hours. She would frequently go to bed well, and rouse her mother before midnight in a hard convulsion; or, she would be playing and suddenly take a spasm; or, she would become angry, throw herself upon the floor and take a spasm; indeed, any unusual little incident might produce a spasm. The child is quite fleshy, hence very heavy upon her feet, clumsy and tottering. Her spasms most frequently commenced with a scream or grunt, then at once followed hard jerking of arms, head, trunk and limbs, in rapid succession, presenting, batting of the eyes, with twitching of the muscles of the entire face, frothing at the mouth, and occasionally biting the tongue. The extensors and flexors were both in active operation for several minutes, then a slight subsidence, not an entire abatement. In this remission the pulse became normal, the face natural, and the limbs relaxed; but in a moment, the same general contortion was enacted over again. This alternation continued for nearly two hours in her last attack, some two months ago. In several of her attacks, hemiplegia made its appearance, but in none so hard as in the last attack. The right side of the face was flushed from the beginning, while the left side was pale. As soon as the spasm entirely disappeared she dropped into a very pleasant sleep. The breathing, which had been hurried, short and stertorous, now became perfectly normal. On waking, which she did in about a half-hour, she called for water, and instead of taking both hands to the glass, as is usual, she applied her right, leaving her left motionless by her side. Her bladder and bowels were both evacuated during the spasm, and now again showed signs of repetition, when her mother took her from the cradle. In doing so, I noticed the left leg was as lifeless as the arm. This hemiplegic condition continued for some ten days, before it entirely disappeared. Belladonna 30th had always before done the work quickly and well, but in this attack it failed me; but the 200th gave prompt relief, carrying off the epileptic hemiplegia as well. The child now seems perfectly well, and has gone fully thrice as long without a spasm as she has done at any time since their commencement.

This case presented many worm symptoms, such as circumscribed red cheeks, white around the nose, swelled upper lip, picking the nose, pouched navel, distended bowels and variable appetite, and yet, as far as observation went, she never passed any, notwithstanding efficient means had been frequently used. This case clearly proves that worms are often charged for producing symptoms which really originate in, and are disseminated from the nervous centres, independent of worms.

Another case, just the opposite of the foregoing, was that of a child from parents of very similar temperaments, of a sanguineous, mercurial character. The child was of a scrofulous diathesis, very large head, forehead or cerebrum disproportionately large, trunk and extremities thin and lax; always pale, peevish and restless. The mother gave no milk, hence the child was fed from the bottle. Its teething period was greatly postponed. Its mind was all alive, quick to catch an idea, daily added words to its vocabulary, and precociously learned to combine them, and often saying things far beyond its age. When about thirteen months of age its first teething symptoms appeared. On the 10th of last December, the child became unusually ill, mentally, scolding, crying, chattering rapidly against everybody who attempted to pacify it, refused its food, threw away its choice dolls and other playthings, refused all familiarity with every one save its mother, and tolerated her only so far as she permitted her to have her own way. Fever gradually increased until 4 P. M., when a severe clonic spasm set in, wrecking her entire organism. Having been otherwise engaged, I did not see the child until 7 P. M. Found her in one which seemed to affect the nerves and muscles of the head and chest far more than the extremities. The nerves of the face, particularly the trifacial, jerked and twitched exceedingly; her eyes and mouth were in constant motion; the lungs were greatly oppressed, the heart going at the rate of 150 per minute; the eyes dilated and rolled up, and more or less reddened; abdomen bloated and rumbling; mouth in rapid sucking motion, thrusting out the tongue with a clucking noise. She rolled her head jerkingly, with an accompanying moan, and sometimes a piercing shriek, even when she was otherwise comparatively easy. I gave her Hyoscyamus 3x, with some relief; but this only temporary. The brain, no doubt, was disorganized during the first hard spasm. The force of the convulsions gradually waned upon the extremities, but continued upon the brain and chest as long as life remained, which yielded at 11 P. M. Thus ended a precocious life, from want of structural balance, too much medullary and too little muscular substance; too much mental and too little physical—no doubt the legitimate result of an improper marriage. Two persons of analogous temperaments, or of equally scrofulous or syphilitic diathesis should never come together as husband and wife, as their offspring will invariably prove failures. Where only one parent is affected with a scrofulous, syphilitic or mercurial diathesis, then there will be a strong probability that the healthy parent will control the state of the progeny, and bring forward well-developed organisms.

Another case of convulsions occurred in a child of a nervo-bilious temperament. The child passed through all its teething handsomely, until it reached its period for the cutting of its stomach and eye-teeth, when it became very much disturbed by lumbricoides and occasional ascarides; both kinds of which, it frequently passed spontaneously. During the month of August, 1877, the child had its first spasm, under the care of one of my brother-would-be-homœopaths; who, in his love for examining all things and disposition to hold to nothing, gave her an ordinary apothecary shop in the course of a year, without the least relief, but upon the contrary, entailed upon her a multiplicity of symptoms too tedious to enumerate, too vexatious for comfort.

The doctor finally pronounced the child incurable; saying, the child would finally become idiotic, as the spasms had assumed an epileptiform character. In this dilemma I was called in. Found the child in a hard tonic spasm, muscles hard and severely contracted, head drawn back, knees drawn up, throwing her hands and limbs about lawlessly, and vigorously extending them, then drawing them up suddenly. Bowels rumbling and tympanitic; sore to the touch and quite yellow.

I also learned that she had from three to ten spasms in the course of twenty-four hours, and that this condition of things had been repeated every eight or ten days for at least one year. Spasms were liable to come on at any time, day or night, asleep or awake. She breathed like an infant troubled with diaphragmetis, short, labored and grunting, even during her best periods of freedom. I gave her Opium during the spasms, the 30th dilution at first, but changed it for the 200th before the attack was over. Then, through the interim, I gave her Calcarea carb.$^{2c}$, every night a dose. This softened her bowels and removed both the soreness and tympanism, and also the rumbling to a great extent. The spasms returned in fifteen days, several days later than usual, with but little variation from past exhibitions. I gave her Opium$^{2c}$ at once, as she gasped so much for breath. Breathed so loud and heavy, with mouth wide open most of the time, and arms spread straight out, at right angles with body. The medicine acted beautifully, as she had but two, at intervals of five hours.

I gave her Calcarea carb., ad interim, again, and ordered her a daily enema, composed of two tablespoonfuls of Orleans molasses, five drops of spirits of turpentine, and one pint of warm rain-water. This greatly aided regular action of the bowels, and brought away great quantities of worms, alive and struggling. The next attack came on in just four weeks from the last. In this, she was taken during her first nap, about 9 P. M.

Her head, eyes, face and mouth remained quiet, but the body, arms and legs worked as usual. Her bowels moved during the severity of the spasm; she also passed urine. Seemed more troubled in diaphragm than during the two last attacks. It was in every sense of the word, a very decided clonic spasm. I gave her Belladonna$^{2c}$, which cut it short, and she had no more for the present.

The quantity of worms passed during the last attack was really incredible; fully a pint, and these chiefly ascarides, mixed with several unusually large lumbricoides.

From this time onward, the child gained in strength, appetite, general appearance and animation steadily; showing no symptoms whatever of spasms for more than three months, when she contracted a very severe cold, affecting her head, throat and chest, producing most violent fever, and finally one hard spasm. This spasm was far more energetic upon her head than elsewhere. Her head jerked backward and sidewise, with rapid snapping of the eyelids, twitching of the muscles of the mouth, with occasional thrusting forward of the tongue, accompanied with a blowing sound, as though she intended to eject some offensive material from the mouth. All the muscles and nerves of the face twitched or quivered very much; her arms and chest seemed in constant spasmodic action, backward, forward and sidewise. Sometimes the body would be thrown violently backward, and in another moment, would be thrown equally as violently forward; and yet it could not, with propriety, be called opisthotonus, neither could it be called emprosthotonus action, as it was but momentary, in either direction.

Aconite[3] was given for the fever, and continued until the hardest of spasms presented itself, when Hyos.[2c] was given every ten minutes, until all traces of the spasm subsided. The action of the heart was absolutely alarming, as it beat with such force and rapidity, that I could not count them satisfactorily. The whole chest shook from the extreme action of the heart. I feared disorganization, but with the spasm the fever subsided. It was the culmination. Quiet soon reigned. The great storm of arterial orgasm was over, and my little patient again herself. Hyos.[2c] was given once each week for about three months, after which all medication was discontinued. More than three years have now elapsed since her last spasm. She is therefore, no doubt, radically cured of spasms. She is now the pride of the household; active, happy, energetic, and as mentally bright as any child of her age.

I might multiply such cases indefinitely, but I forbear for the present. It is full of the deepest interest to every physician to know every possible symptom of spasms, and every means calculated to cure them; hence this little mite to aid in the great work of promoting the desirable end.

